# Impact of COVID-19 on post graduate medical education and training

**DOI:** 10.1186/s12909-021-03019-6

**Published:** 2021-11-16

**Authors:** Sharon C. Enujioke, Kimberly McBrayer, Katherine C. Soe, Teresa M. Imburgia, Cynthia Robbins

**Affiliations:** 1 Division of Adolescent Medicine, Department of Pediatrics, Indiana University School of Medicine, 410 West 10th Street. Suite 1001, Indianapolis, IN 46202 USA; 2 Department of Pediatrics, Indiana University School of Medicine, Indianapolis, IN USA; 3 Department of Psychiatry, Indiana University School of Medicine, Indianapolis, IN USA; 4 Indiana University School of Social Work, Indianapolis, IN USA

## Abstract

**Purpose:**

The novel Coronavirus Disease 19 (COVID-19) has had a significant impact worldwide that led to changes in healthcare. The purpose of this study was to evaluate the effect of the COVID-19 pandemic on trainee’s mental health and educational preparedness.

**Methods:**

Trainees at the Indiana University School of Medicine were surveyed regarding the impact of the COVID-19 pandemic on their training. Using a Likert scale, participants were asked questions pertaining to educational preparedness, mental health, and clinical work during the pandemic. Data was analyzed using SPSS version 27. The study was approved as exempt by the Institutional review Board (IRB).

**Results:**

324 of the 1204 trainees responded to the survey. The respondents were 76% white with an equal distribution of males and females. A majority of the respondents were first year residents with an equal distribution of second, third, and fourth year residents. Twenty-three percent of respondents were in a procedural residency or fellowship program. Better perceived educational preparedness was associated with an improved home-work balance during COVID-19 (β = 0.506, *p* < 0.0001) and having a department that advocated/supported focus on mental health during COVID-19 (β = 0.177, p < 0.0001). Worse perceived educational preparedness was associated with being in procedural vs. non-procedural dominant training program (β = − 0.122, *p* = 0.01).

**Conclusion:**

COVID-19 has had a significant impact on the training experience of residents and fellows. Departmental support increased mental well-being and perceived education preparedness in trainees. Trainees that felt they had a better home-work life balance had better educational preparedness compared to their peers. Also, trainees in procedural programs had less educational preparedness compared to their peers in non-procedural programs. This study highlights the importance for programs to find avenues to increase educational preparedness in their trainees while being attuned to the mental health of their trainees.

## Introduction

The novel Coronavirus Disease 19(COVID-19) has had a significant impact on health care systems around the world. The COVID-19 pandemic’s effect on resident physicians and fellows in training remains limited. Studies of residents during this time have examined the use of telemedicine as a means of promoting safety while maintaining access and minimizing disruption in clinical care. Many innovative solutions have arisen in response to medical and surgical education changes, including virtual learning, videoconferencing, and social media [1]. However, these have also highlighted limitations with regard to rapport development with patients, privacy and confidentiality, and access to technology [2].

One study of resident mental health at a large academic medical center revealed significantly higher rates of reported stress and burnout in COVID-19 exposed physician trainees compared to non-exposed. Female and unmarried trainees demonstrated higher rates of stress and depression, respectively [3]. In fact, over half of resident trainees in Brooklyn, New York expressed anxiety for at least several days over a two week period [4]. Despite decrease in operating room (OR) time, a survey of otolaryngologists during the COVID-19 pandemic found that nearly half reported anxiety, depression, distress and burnout, with a greater prevalence in residents (compared to attendings), females, and those in states with over 20,000 positive cases [5]. Together, these studies reflect a common theme: the importance of being mindful of and supporting the mental health of frontline healthcare workers, including physician trainees.

To our knowledge, this is the first study to assess the perceived impact of the COVID-19 pandemic on residents’ and fellows’ clinical training and mental health support across multiple subspecialties at a large medical institution. This study aims to describe the impact of COVID-19 on post-graduate medical trainees and investigate what was associated with positive educational preparedness during COVID-19.

According to the Indiana State Department of health, there have been over 672 K COVID cases with 12 K deaths in Indiana, representing the thirteenth highest death rate in the country [6]. The highest rate of COVID admissions and deaths occurred in December of 2020 [7, 8]. Indiana University Health (IUH) is the largest network of physicians and hospitals in the state. IUH partners with the Indiana University School of Medicine (IUSOM), which sponsors 100 ACGME-accredited residency and fellowship programs. In 2021, there were 985 residents and 219 fellows associated with IUH and IUSOM [9, 10]. The COVID vaccine was available to all house staff at the time of this survey.

## Methods

### Participants

Participants were sampled from the 1204 residents and fellows associated with IU School of Medicine. They received an emailed survey to complete. This represented all residents and fellows across all programs at IUSOM.

### Materials

Participants were asked demographic data, overall impression of training during COVID-19 and questions pertaining to educational preparedness, clinical work, and mental health during the pandemic. Participants could leave comments on the final question. Gender, race, marital status, whether they had children living at home, and COVID-19 exposure and if they acquired the infection status were assessed. Overall impression included questions such as “COVID-19 has had a positive impact on my training.” Educational preparedness included “I have had more time for self-directed study during COVID-19” and “I feel more prepared for my Inservice training exam (ITE)/boards.” Questions were measured on a Likert type scale of 1 through 5, with 1 being strongly disagree and 5 being strongly agree.

### Procedure

The survey was implemented via a web-based platform (Redcap) and completed by means of voluntary participation. Surveys were distributed by email through the Graduate Medical Education (GME) office at the beginning of March 2021 and re-sent three weeks later to all trainees. The team then reached out to program directors to email the survey to their trainees. After a month of collecting responses, the survey was closed for analysis of results. The individual email addresses were not known by the researchers, and data was de-identified by Redcap.

Data analysis was performed with SPSS version 27 for Windows (IBM, Armonk NY). Frequency data are presented on demographics and COVID-19 impact. Due to lack of power, transgender individuals were coded with their associated gender (e.g. transgender females coded with cis-gender females) and two non-binary individuals were coded as missing for the purpose of analysis involving gender. Racial data was analyzed both with individual races and with races grouped as white and non-white. Fifty-two different resident and fellow groups answered the survey, and these were grouped into procedural (determined as all procedure-dominated residencies and fellowships) and non-procedural programs for added power. Procedural program included all surgical specialties and subspecialties as well as anesthesiology, dermatology, and pediatric dentistry.

Five educational items were combined into an educational preparedness scale (Chonbach’s alpha = 0.61) to assess what items were associated with a better perceived educational preparedness during COVID-19. The study was approved as exempt by the Indiana University Institutional Review Board (IRB).

### Statistical analysis

Bi-variate associations of candidate predictors with perceived educational preparedness were analyzed using t-tests and chi-squared tests. Predictors were then entered into a multivariable linear regression model.

## Results

The survey was sent to the 1204 trainees at IUSOM and 324 completed the survey. Of the respondents, 48% identified as female and 76% as white. Sixty-four percent identified having a current partner, and 20% had at least one child under the age of 18. Mean years post-graduate were 2.9 (1.6), with a majority of the respondents identifying as first year residents and an equal distribution of the second through fourth year residents. Twenty-three percent of respondents identified as being involved in a procedural residency, which was classified as a procedural dominant specialty or fellowship program. Positive COVID-19 testing in the trainee or their immediate household was 15%; 31% of respondents endorsed having to quarantine at least once. Mean answer for the Educational preparedness domain questions was 3.0 (0.7). Table 1 provides demographic information and Table 2 provides the mean scores of overall COVID impact, educational preparedness, clinical questions, and mental health items. Table 3 provides bivariate analysis involving education preparedness questions compared to demographic data and mental health questions, which yielded significant results for female gender (3.1 vs. 2.9 for males, *p* = 0.001), number of years post-graduate (*p* < 0.0001), no kids (3.1 vs. 2.8 for those with kids, *p* = 0.003), and departmental support of mental health (2.2, 0.9, p < 0.0001). Multivariable analysis for educational preparedness was performed using linear regression (see Table 3). The adjusted R^2^ for this model was 0.364. Better perceived educational preparedness was associated with an improved home-work balance during COVID-19 (β = 0.506, *p* < 0.0001) and having a department that advocated/supported focus on mental health during COVID-19 (β = 0.177, p < 0.0001). Worse perceived educational preparedness was associated with being in procedural vs. non-procedural training program (β = − 0.122, *p* = 0.01).

**Table 1:** Sample characteristics (*N* = 324)

	Mean, SD or n, %
Gender female	150, 47.5%
Race white	221, 75.9%
Has a current partner	204, 63.7%
Procedural dominated programs*	67, 20.6%
Has Kids	66, 20.4%
First year resident	85, 26.3%
Number of years post grad	2.9, 1.6
You or someone in your immediate household tested positive for COVID	50, 15.4%
Quarantined for COVID contact	100, 30.9%

*General surgery, orthopedic surgery, anesthesia, dermatology, otolaryngology, OBGYN, Urology, OMFS, peds dentistry, urogynecology, transplant nephrology, uro-endourology, neurosurgery, ophthalmology, peds urology, peds cardiac anesthesia

**Table 2:** COVID Impact on education, clinical skills, and mental health

COVID Items (1 = strongly disagree – 5 = strongly agree)	Mean, SD
***Overall COVID attitude***	
COVID has had a Positive Impact on training	3.56, 1.1
COVID has had a Negative Impact on training	2.02, 0.82
***Educational Attainment (5 items, α = 0.641)***	**3.0, 0.7**
More time for self-directed study	2.95, 1.11
Felt prepared for ITE	3.54, 0.85
Zoom lectures were effective	3.44, 1.29
Preparedness to practice	2.31, 0.97
I have had time to read	2.78, 0.91
***Clinical Items***	
Efficient at research	2.95, 0.99
Clinical skills not impacted by COVID	3.13, 1.14
Have time to care for patients	2.99, 0.92
***Mental Health Items***	
Improved life-work balance	3.60, 1.10
Had to focus more on taking care of mental health	2.34, 1.07
Department advocated/supported focus on mental health	2.24, 0.874
Able to cope with changes	2.75, 0.99

**Table 3:** Bivariate and Multivariate Analysis for Educational Attainment

	Educational attainment Mean	Bivariate Beta	*P* value	Multivariable Standardized Beta^a^	P value
Gender:					
Female	3.1	0.177	0.002	1.589	0.068
Male	2.9				
Race:					
White	3.0	0.086	0.145	0.820	0.413
Non-white	2.9				
Current partner	2.99	−0.012	0.829	0.051	0.311
No current partner	3.01				
Surgical	2.8	−0.114	0.085	−0.122	0.012
Non-surgical	3.0				
Has Kids	2.8	−0.164	0.003	−0.089	0.085
No Kids	3.1				
Improved life-work balance.	–	0.566	< 0.0001	0.506	< 0.0001
Had to focus more on taking care of mental health	–	−0.006	0.991	0.002	0.973
Department advocated/supported focus on mental health	–	0.291	< 0.0001	0.177	< 0.0001

^a^*R*^2^ = 0.363

## Discussion

This study adds to a growing body of literature on the impact of the COVID pandemic on physicians in training (resident physicians). The COVID pandemic created unique challenges for all of healthcare, and many institutions rely on physicians in training to staff hospitals and clinical programs, especially during times of high clinical volumes. The pandemic also created unique learning opportunities, with some disciplines more impacted than others based on the needs of COVID patients. Our survey found a relationship between better work life balance and positive education preparedness during the pandemic. Resident physician wellness is increasingly emphasized as a priority for training programs and mandated by the ACGME common program requirements; quality of life factors like sleep and social connection as well as educational autonomy and competence building have been found to be related to wellness [11].

Residency programs should have firm protections built in their design to protect quality of life which withstand stresses put on their clinical programs. Wellness positively impacts educational preparedness. Other studies have found preserving safety (personal protective equipment access and protection against contracting the virus) and maintaining high quality education can impact resident stress, so these are important areas to focus on for wellness. These studies also found transparency in program changes was also related to resident stress, and resident input into program changes should also be employed [12–15].

Our results also suggest that an individual’s perceived departmental mental health support significantly correlated with educational preparedness during the COVID-19 pandemic. Although previous studies have examined mental health effects of the pandemic and changes in education for trainees, this is the first to our knowledge to report a correlation between these two variables. A systematic review examining challenges and innovative solutions for trainee education during the pandemic highlighted the rise in virtual solutions, including video conferencing platforms for both patient care and education, as a means of countering the limitations imposed by pandemic-related restrictions [1]. Principle challenges identified in the same review were largely related to the shortage of personal protective equipment (PPE) and drop in elective surgical cases. Both of these affect surgical residents more than non-surgical. This lack of PPE and reduction in resident-led elective surgery cases is likely to have affected perceived departmental support as well. A survey of urology residents across the United States further identified risk factors of worse mental health outcomes, which included perceived PPE access problems and susceptibility of household members, and local COVID-19 severity [16]. These studies both support and may help explain why surgical residents perceived a greater educational detriment during the pandemic than those who could utilize virtual means to assist with their assessments and management.

Notably, studies have consistently found that resident physician anxiety during this period often centered around loved ones’ health over their own [16, 17]. One study found that the greater level of stress and burnout reported by those exposed to COVID-19 positive patients often included a cascade of worries about childcare and work-family balance [3]. Thus, it is reasonable that those who felt better supported in these domains would then have more reserve to engage in their education.

Recognizing the importance of frontline worker mental health, many programs both in and outside of the IU School of Medicine established new mental health access programs for healthcare provider assessments and therapy. Given that awareness is key to utilization, it would be interesting to investigate whether awareness of this program at the study institution affected perceived departmental support. Many programs also held virtual social gatherings in attempts to encourage social support systems. Telehealth peer support for frontline clinicians in the form of group and individual counseling facilitated by videoconferencing and phone, specifically regarding their work with COVID-19 patients, has been studied with reported utility in emotional processing, validation, and peer learning [18].

During the COVID-19 pandemic, residency and fellowships programs were faced with the task of restructuring their programs to protect trainees from contracting COVID-19 while maintaining their educational preparedness. While medical specialties rapidly evolved to telehealth visits, this could not replace the hands-on experience that procedure-dominated specialties glean from working in the operating room. This issue was reflected in our study findings, as non-procedural trainees reported higher levels of perceived educational preparedness compared to trainees in procedural programs during the study period. Despite reduction in operating room time, surgical trainees reported increase in clinic and telemedicine visits leaving less room to study as well increased work burden when a fellow trainee was quarantined. Surgical residents had a significant decline in case volume during the pandemic. While the American Board of Surgery (ABS) modified graduation requirements, residents felt uncomfortable graduating with less than the traditional number of required cases. Similar to medical training programs, surgical programs adapted to COVID-19 by virtual didactics and telehealth visits. A few programs incorporated surgical simulators [19]. While telemedicine visits and didactics can offer some educational benefit, there is a push toward simulation training that replicates safety scenarios and targets training gaps due to the decreased workload during COVID-19 [20]. Looking at tools used during COVID-19 pandemic to ensure trainees had optimal education preparedness at different programs will be imperative in planning for future pandemics. Key recommendations gleaned from our study are listed in Table 4.

**Table 4:** Key Recommendations

• Programs should protect work-life balance by limiting work obligations during off hours	
• Mental health interventions to support wellness and treat problems should be normalized in residency and integrated into all residency programs	
• Provide support for trainees who have children to include access to daycare, time off when a loved one is sick, or supporting schedule changes to accommodate unique needs	
• Social connectedness amongst residents can be integrated into programs via planned activities that are safe when social distancing is important, such as book clubs or outdoor meet ups	
• Residencies requiring hands on skills (such as surgical residencies) should have	
• simulation or lab back up opportunities for procedure learning	

### Limitations

A limitation of this study was that specific components of well-being was not evaluated. Also, there was no pre-COVID evaluation of training experience. Given the many training programs in the institution, we did not ask what changes had been implemented in each program to help with mental health during the COVID-19 pandemic. Also, due to the relatively low response rate, there may have been some degree of selection bias.

## Conclusion

COVID-19 has had a significant impact on the training experience of residents and fellows, and this impact will be long-lasting. As a result of the pandemic, programs at our institution converted education online, held online social events, and integrated mental health support into their programming. Programs continue to be tasked to find ways to increase educational preparedness for trainees while ensuring attention is paid to the mental well-being of the trainees. Opportunities to increase clinical learning without overwhelming trainees is a balance that all program face. Lessons from this pandemic should be used to glean ways to improve training and education for residents in the event of another pandemic or restriction of in-person learning.

## Data Availability

The datasets used and/or analyzed during the current study are available from the corresponding author upon request.

## Abbreviations

CMMS: Centers for Medicare and Medicaid Services
OR: Operating Room
ACGME: Accreditation Council for Graduate Medical Education
IUH: Indiana University Health
IUSOM: Indiana University School of Medicine
GME: Graduate Medical Education
ITE: In- training exam
IRB: Institutional Review Board
PPE: Personal Protective Equipment
ABS: American Board of Surgeons
